# Deep Learning in Drug Discovery and Medicine; Scratching the Surface

**DOI:** 10.3390/molecules23092384

**Published:** 2018-09-18

**Authors:** Dibyendu Dana, Satishkumar V. Gadhiya, Luce G. St. Surin, David Li, Farha Naaz, Quaisar Ali, Latha Paka, Michael A. Yamin, Mahesh Narayan, Itzhak D. Goldberg, Prakash Narayan

**Affiliations:** 1 Department of Preclinical Research, Angion Biomedica Corporation, Nassau, NY 11553, USA; ddana@angion.com (D.D.); sgadhiya@angion.com (S.V.G.); lstsuri1@jhu.edu (L.G.S.S.); david_li@college.harvard.edu (D.L.); fnaaz@angion.com (F.N.); qali@angion.com (Q.A.); spaka@angion.com (L.P.); myamin@angion.com (M.A.Y.); igoldberg@angion.com (I.D.G.); 2 Department of Chemistry and Biochemistry, The University of Texas, El Paso, TX 79968, USA; manarayan@utep.edu

**Keywords:** drug discovery, therapeutics, small molecules, precision medicine, artificial intelligence, deep learning, transfer learning, recurrent neural networks, de novo design

## Abstract

The practice of medicine is ever evolving. Diagnosing disease, which is often the first step in a cure, has seen a sea change from the discerning hands of the neighborhood physician to the use of sophisticated machines to use of information gleaned from biomarkers obtained by the most minimally invasive of means. The last 100 or so years have borne witness to the enormous success story of allopathy, a practice that found favor over earlier practices of medical purgatory and homeopathy. Nevertheless, failures of this approach coupled with the omics and bioinformatics revolution spurred precision medicine, a platform wherein the molecular profile of an individual patient drives the selection of therapy. Indeed, precision medicine-based therapies that first found their place in oncology are rapidly finding uses in autoimmune, renal and other diseases. More recently a new renaissance that is shaping everyday life is making its way into healthcare. Drug discovery and medicine that started with Ayurveda in India are now benefiting from an altogether different artificial intelligence (AI)—one which is automating the invention of new chemical entities and the mining of large databases in health-privacy-protected vaults. Indeed, disciplines as diverse as language, neurophysiology, chemistry, toxicology, biostatistics, medicine and computing have come together to harness algorithms based on transfer learning and recurrent neural networks to design novel drug candidates, a priori inform on their safety, metabolism and clearance, and engineer their delivery but only on demand, all the while cataloging and comparing omics signatures across traditionally classified diseases to enable basket treatment strategies. This review highlights inroads made and being made in directed-drug design and molecular therapy.

## 1. Introduction

Human civilization has a lofty record of its efforts to fight disease and ailments. Based on local and prevalent dogmas, civilizations developed and adopted unique ways to treat the patient. Thousands of years ago the field of drug design and molecular therapy was defined by a more holistic approach with the focus on both the mind and body of the ailing, not just the disease alone. This approach yielded Ayurveda in India, Unani medicine in the Middle East and traditional Chinese medicine (TCM) in the Far East. Many of these practices continue to carry favor today with governmental agencies including the Department of Defense and the National Institutes of Health (NIH)/National Center for Complementary and Integrative Health funding research to determine best practices across a spectrum of traditional medical practices from acupuncture to yoga. Nevertheless, the last century saw allopathy being adopted as the prevalent medical practice across the globe. In fact, in the Western world, allopathy is the norm. With its advent in the nineteenth and twentieth centuries, the American Medical Association was created and the pharmaceutical industry began to rise as antibiotics were synthesized. Allopathic practitioners seek to treat disease by examining and attacking the physical causes, whether external or genetic and seeks to produce effects that suppress or eradicate symptoms of the disease. A vast amount of empirical evidence speaks to the success of this approach in healthcare. Also, the technologies developed, especially inroads made in imaging, visually pinpoint the cause of the disease, greatly aiding in the treatment of both acute and chronic diseases. 

## 2. Precision Medicine

Despite the remarkable success of allopathy, post-marketing surveillance data show that its benefit is not experienced by every patient. Patients on the same drug can also respond differently. This phenomenon is most evident during the conduct of highly controlled Phase II and III clinical trials when there is more often than not a differential response to the experimental drug [1,2]. Results from numerous trials show that despite judicious selection of inclusion and exclusion criteria, within the “active” cohort a subset of participants meets the primary endpoint whereas another subset does not [3]. Such findings coupled with the genomics revolution has spurred the use of precision medicine for treating a patient [4,5]. Technological advances, coupled with the shrinking cost of genome sequencing and analysis, characterized the genomics revolution. Because researchers are now able to sequence all the deoxyribonucleic acid (DNA) of an organism and its genome, it is possible to compare the genomes of different organisms and populations [6]. The genomics revolution can impact on how patients will react to drugs. Often overlooked, the concept of precision medicine took root during blood transfusion and was then adopted in solid organ transplantation. Precision medicine is becoming the norm with clinical decisions based on genomic information. By identifying the genomics of patients and their diseases scientists are now able to make certain treatment decisions based on whether a drug will be effective, ineffective or toxic [7]. Physicians can thus spare potential non-responders the effort, cost and risks of taking a drug that is unlikely to prove beneficial in those patients—the goal being to deliver the right medicine to the right patient every time. Under the administration of Barack Obama, a nationwide precision medicine initiative was launched in 2015 seeking to individualize treatment and prevention strategies for diseases through federally supported research initiatives [8]. Rather than the one-size-fits-all method, healthcare professionals in several disciplines are now treating patients based on factors including their genetic makeup, where they live, and the lifestyle they practice—i.e., a panomic approach which takes into account both the interactome (genome+transcriptome+proteome) and the exposome. Cancer patients have been by far the largest beneficiaries of precision medicine. Cancer usually is a result of gradual accumulation of genetic changes (often called mutations) in genes that control cell growth. In this sense cancer is very much a disorder of the genome. Depending on where in the body the cancer arises and the types of genetic changes the cells accumulate, different types of cancer can have very different genetic profiles. These genetic profiles can be used in a number of ways to aid doctors in choosing the best treatments for each individual patient. By comparing the DNA from a patient’s tumor to that of their normal cells [9], researchers can learn how the cancer arose and where it may be vulnerable to treatment. Some precision medicine cancer treatments already in use target specific molecular markers that are found only on certain types of cancer. For example, colon cancers that have a normally functioning version of a surface protein called KRAS are likely to respond to certain anti-epidermal growth factor receptor (EGFR) antibody therapies; those in which the protein is absent or non-functional are not. Two other targeted cancer treatments currently in use are Herceptin and Opdivo [10,11]. In addition to improving outcomes with treatments of certain cancers [12], leveraging of the precision medicine platform has started influencing other spheres within medicine. For example, it has been discovered that a subset of patients with highly aggressive rheumatoid arthritis exhibits autoantibodies to the neutrophil nuclear enzyme peptidyl arginine deiminase 4 (PAD 4) [13]. These autoantibodies are active in that they exacerbate disease. The pharmaceutical industry is targeting this subset of patients with orally bioavailable small molecule PAD 4 inhibitors. The presence of citrullinated histones and/or citrullinated proteins in patients with acute tissue injury represents another beacon for intervention with a PAD 4 inhibitor in that population [14]. Epigenetic therapy is the use of drugs or other epigenome-influencing techniques to treat medical conditions. Many diseases, including cancer, heart disease, diabetes, and mental illnesses are influenced by epigenetic mechanisms, and epigenetic therapy offers a potential way to influence those pathways directly. Diabetic retinopathy is known to be associated with a number of epigenetic markers, including methylation of the superoxide dismutase 2 (Sod2) and matrix metalloproteinase-9 (MMP-9) genes, an increase in transcription of Lysine-specific histone demethylase 1 (LSD1), a H3K4 and H3K9 demethylase, and various DNA Methyl-Transferases (DNMTs), and increased presence of microrubonucleic acids (miRNAs) for transcription factors [15,16]. Several avenues to epigenetic treatment of diabetic retinopathy have been studied. One approach is to inhibit the methylation of the Sod2 and MMP-9 [17]. The DNMT inhibitors 5-azacytidine and 5-aza-20-deoxycytidine have both been approved by the Food and Drug Administration (FDA) for treatment of other conditions, and studies have examined the effects of those compounds on diabetic retinopathy, where they seem to inhibit these methylation patterns with some success at reducing symptoms [17]. The DNA methylation inhibitor Zebularine has also been studied, although results are currently inconclusive [18]. A second approach is to attempt to reduce the miRNAs observed at elevated levels in retinopathic patients, although the exact role of those miRNAs is still unclear. The Histone Acetyltransferase (HAT) inhibitors Epigallocatechin-3-gallate, Vorinostat, and Romidepsin have also been the subject of experimentation for this purpose, with some success [19,20,21]. Cancer epigenetics is the study of epigenetic modifications to the DNA of cancer cells that do not involve a change in the nucleotide sequence. Epigenetic alterations may be just as important, or even more important, than genetic mutations in a cell’s transformation to cancer. Epigenetic control of the proto-onco regions and the tumor suppressor sequences by conformational changes in histones plays a role in the formation and progression of cancer. Pharmaceuticals that reverse epigenetic changes might have a role in a variety of cancers. Recently, it has become evident that associations between specific cancer histotypes and epigenetic changes can facilitate the development of novel epi-drugs [22]. Drug development has focused mainly on modifying DNMT, HAT and histone deacetylase (HDAC). Drugs that specifically target the inverted methylation pattern of cancerous cells include the DNA methyltransferase inhibitors azacitidine and decitabine [23,24]. These hypomethylating agents are used to treat myelodysplastic syndrome, a blood cancer produced by abnormal bone marrow stem cells. These agents inhibit all three types of active DNA methyltransferases, and had been thought to be highly toxic, but proved to be effective when used in low dosage, reducing progression of myelodysplastic syndrome to leukemia [23]. HDAC inhibitors show efficacy in treatment of T cell lymphoma [25]. Two HDAC inhibitors, vorinostat and romidepsin, have been approved by FDA.

**Figure 1:**
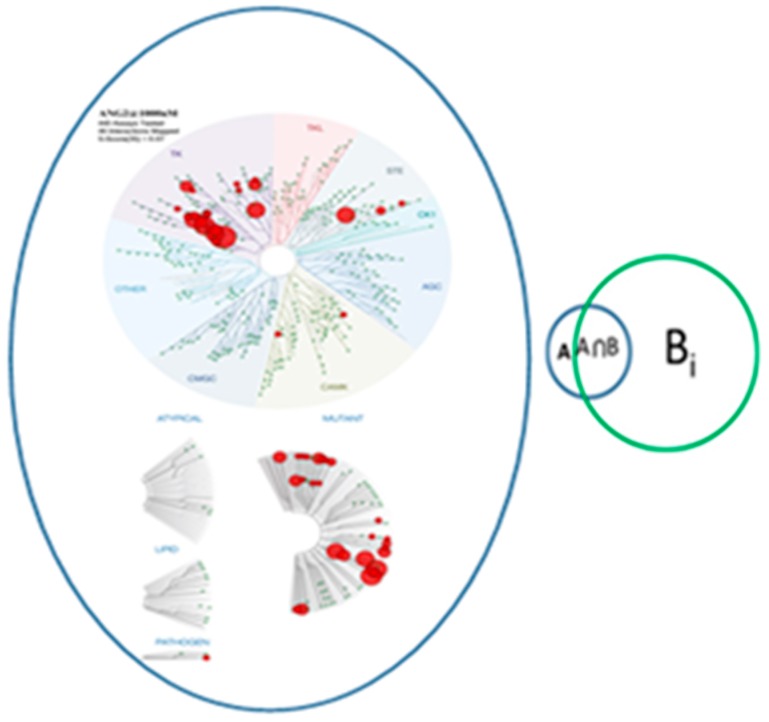
**Precision medicine in kidney disease.** The signalosome of ANG3070, an orally bioavailable small molecule fibrokinase inhibitor is depicted within the blue circle. The red hotspots indicate signaling elements modulated by this drug candidate. A precision medicine approach is being used to inform inclusion criteria for an ANG 3070 clinical trial in nephrotic syndrome- focal segmental glomerulosclerosis (NS-FSGS). Renal biopsy and urinalysis from these patients will be subjected to omics analysis and signalosomes (green circle) identified for each patient. Only those patients will be recruited that share 70% of the ANG3070 signaling elements.

Use of precision medicine is making major inroads in nephrology. The Nephrotic Syndrome Study Network, NEPTUNE is implementing the concept of precision medicine for the development of new disease definitions to be informed by a comprehensive, multilayered analysis of the disease course in observational cohort studies [26]. NEPTUNE is recruiting patients with nephrotic syndrome (NS) and has generated datasets which define the underlying genetic architecture and capture environmental exposures, unique molecular phenotypes, histopathology, and prospective clinical outcomes. This disease knowledge along the genotype–phenotype continuum can and is being used by basic and clinical scientists to develop a knowledge network of NS that defines the diseases from molecular pathogenesis rather than from histopathologic patterns [26]. The molecular disease definition (i.e., taxonomy) will allow more accurate diagnosis, which is a prerequisite for targeted treatments that improve health outcomes in NS. In fact, Angion Biomedica Corp. is partnering with NEPTUNE to inform inclusion criteria for a clinical trial of its orally biaoavilable fibrokinase inhibitor ANG3070 in NS-focal segmental glomerulosclerosis (FSGS) based on the drug’s signalosome. The ANG3070 signalosome associated with beneficial effects in preclinical models of disease will be compared with the signalosome of individual FSGS patients (Figure 1). Only those patients that house at least 70% of the ANG3070 disease-modulating signalosome will be recruited in a safety and efficacy trial with this drug. In other words,

(1) $$\;J\;\left(A,B\right)\;\frac{\left\{A\cap B\right\}}{\left\{A\right\}}\geq 0.7=\mathrm{enroll}\;\mathrm{patient}\;$$

The precision medicine platform is also being leveraged to deliver point-of-care diagnosis in patients. As an example, emphasis is now on querying the BALFosome (broncheoalveolar lavage fluid) in patients presenting with acute respiratory distress or urine transcriptome or proteome in patients presenting with acute kidney disease or NS [27,28,29]. Use of a minimally invasive or non-invasive omics query strategy spares the patient pain and/or distress and provides valuable information that can be acquired relatively rapidly, enabling enrollment or exclusion of the patient. Indeed, the next several years will bear witness to unprecedented success stories from precision medicine in a variety of undermet and unmet needs. Finally identification of signaling networks in disease can be used to develop drug discovery and drug repositioning strategies against that disease. A stellar example is the use of Adalimumab in the Novel Therapies for Resistant FSGS (FONT) trial based on experimental and clinical data supporting the role of tumor necrosis factor α (TNF-α) in the pathogenesis of a wide spectrum of kidney diseases including FSGS [30].

In summary, the use of precision medicine can reduce noise in clinical trials, can reduce cost and duration of those trials, can be prognostic and can spur drug discovery or drug repositioning.

## 3. Drug Repositioning 

The cost associated with bringing a new drug to the market can be staggering, up to $2.0 billion [31]. Coupled with cost is time—on average it takes 10–12 years of discovery and developmental work to bring a drug to the market. With a limited patent life on the composition of matter, there is a limited window to recover costs. Repositioning or repurposing involves the use of existing drugs for another indication following verification of efficacy in the new indication. The drug being repurposed may be one that is already being marketed or an abandoned drug. A drug candidate that has been “shelved” after a Phase II trial for lack of efficacy or shelved from a purely business perspective can also be repurposed. In each case, the drug has been proven “safe” in preclinical toxicology studies and through Phase I or Phase I and II safety trials. Repurposing de-risks the program, saves money and time, bypassing several regulatory hurdles. As an example, it is estimated that $40–$80 million was spent on approval of thalidomide for multiple myeloma compared to the $1–$2 billion it would cost to develop a drug from scratch [32]. Many academics have found promise in drugs that have long been on the market, drugs whose patents have expired. Non-profit companies help usher in these discoveries, which lack monetary incentives, to the clinic. Some companies hoping to recoup returns on their investments are also looking to repurpose existing drugs still under patent protection, such as those that were shelved after unsuccessful clinical trials. Because resources have already been devoted to these unapproved therapies, companies see value in attempting to revamp them for new indications. The National Center for Advancing Translational Sciences within the NIH aims to bridge the industry-academia divide by opening pharma’s storehouse of compounds to university researchers for studies of their mechanisms and potential uses [33]. The center, established in December 2011, funded nine drug projects in 2013 and another four in 2015. Ongoing phase 2 clinical trials grew out of these projects and the center announced funding of several new projects in 2017. 

**Figure 2:**
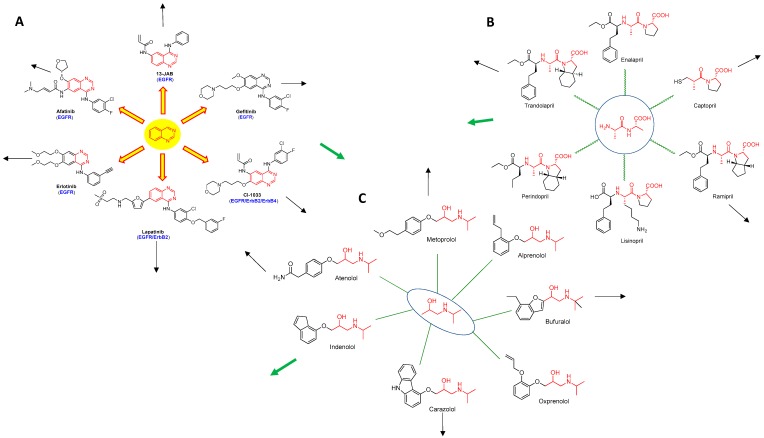
**Erdos interactome representations of drugs.** Many drugs developed for a given indication usually share a common core motif. Substitutions are made on this core scaffold to tune the biological and drug-like properties and generate intellectual property. An Erdos interactome representation for drugs available or designed to treat a particular disease can be built with the drugs closest to the center sharing that core. More distant members in this interactome will more than likely have different cores. At the farthest reaches of this interactome, members might find use in other disease as their structures (green arrows) might resemble or completely overlap with drugs designed for other indications. (**A**) Part of the receptor tyrosine kinase inhibitors interactome centered around a quinazoline core. (**B**) Part of the angiotensin converting enzyme inhibitors interactome centered around an aminopropanamido-propanoic acid core. (**C**) Part of the beta blockers interactome centered around an isopropylamino-propan-2-ol core.

**Figure 3:**
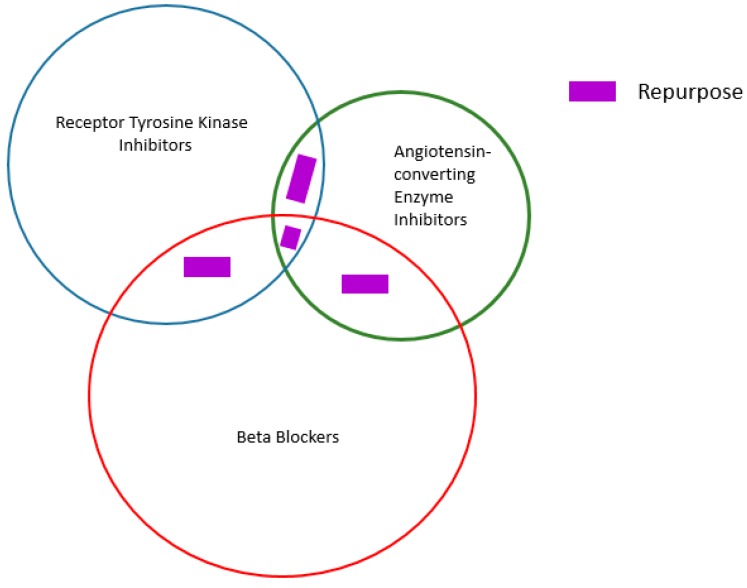
**Algorithm for drug repurposing.** In this example, a Venn diagram comprising Erdos interactomes for receptor tyrosine kinase inhibitors, angiotensin converting enzyme inhibitors and beta blockers is shown. Intersection sets represent drugs that can be repurposed. For example, the intersection set between the red and green interactomes represent drugs or drug candidates that can potentially find use as angiotensin converting enzyme inhibitors or beta blockers.

With repurposing, a key issue is how one knows which indication to repurpose what drug for? Graph theory and network analysis is already in use in a number of areas. As an example this methodology is used in marine biology to compare genetic similarities in geographically distant pods of mammals. The approach proposed herein is to use Erdos Interactomes of drugs to determine whether a drug, called a “node” in this graphical representation (Figure 2), positioned for a given indication can be repurposed for another indication. In this method, drugs are anchored around an original core motif. For receptor tyrosine kinase inhibitors (TKIs), this core is quinazoline. A first order Erdos interactome around quinazoline is shown in Figure 2A. Second order drugs in this interactome might still retain the quinazoline core but host major changes in several side chains. Third order drugs are more loosely related to the quinazoline core as they could be subjected to some core hopping. The outermost belt of this interactome retains TKI properties but bears little resemblance to the core motif or first order drugs. Similar Erdos interactomes have been shown for angiotensin-converting enzyme inhibitors (Figure 2B) and beta blockers (Figure 2C). Shown in Figure 3 is the Venn diagram for these interactomes. Drugs that fall within the intersection sets (green arrows) could potentially find a role in multiple diseases. Given that big pharma has large libraries of compounds, this approach represents a logical and potentially useful scouting algorithm for repurposing drugs.

## 4. Artificial Intelligence (AI) in Drug Design and Molecular Medicine 

Although attractive from the regulatory and business perspectives, repositioning is not always feasible. De novo drug design and synthesis remain the cornerstone of pharmaceutical research and development. The ever-expanding cosmos of molecules has aided drug discovery over decades, however, the enormity of synthesizing all feasible molecules (between 10^60^–10^100^) is a Herculean task. Although the 20th century has been marked by a quantum leap in drug discovery and invention of new therapeutics, the cost and time associated with this endeavor has increased. This is often attributed to multivariable parameters that include synthetic feasibility, in vivo activity, toxicity studies etc. Nevertheless, medicinal chemistry and medicine have started to harness the revolutions in big data, deep learning and artificial intelligence (AI) that are sweeping other disciplines.

**Figure 4:**
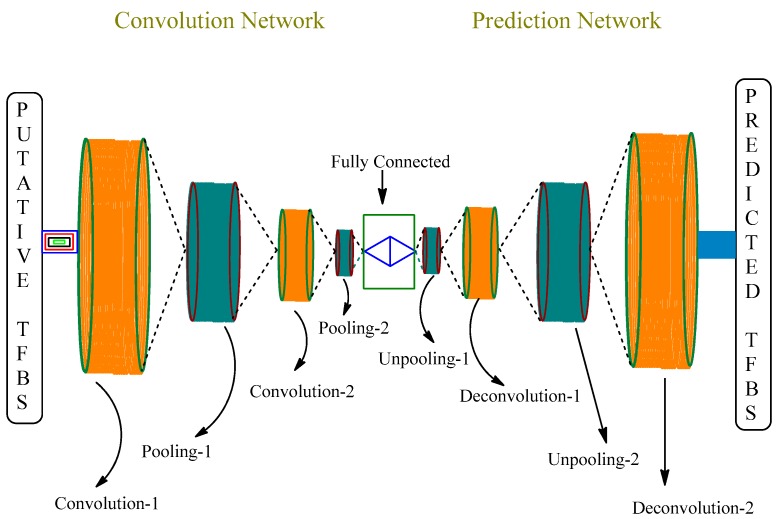
Graphical representation of a DeepSNR model.

One of the challenges in harnessing advances in genomics to therapeutic gain is to decode the multifaceted regulatory system that controls gene expression. Controlled at various stages, regulation of gene expression involves many factors including DNA methylation, regulatory RNAs, and transcription factors (TFs) [34,35]. Among them, the binding of TFs to specific DNA sequences known as transcription factor binding sites (TFBSs) that impart positive or negative control on the transcription of corresponding target genes is a major regulatory component. Revealing and pinpointing TFBSs for a given TF, remains a long-sought goal in applied genomics [36,37] Chromatin immunoprecipitation sequencing (ChIP-seq) assay has been the gold standard for evaluating the interaction of TF with DNA. ChIP-seq, a technology that couples chromatin immunoprecipitation with massively parallel sequencing, is capable of mapping genome-wide TFBSs [38,39]. As with many high-throughput sequencing approaches, ChIP-seq produces enormously large data sets, for which appropriate computational analysis methods are required. To predict DNA-binding sites from ChIP-seq read count data, peak calling methods have been developed. Some of the popular models are MACS [40], ODIN [41], Hpeak [42], QuEST [43], MOSAiCS [44] and many others. Resulting motif data sets are fed in to MatInspector [45] or MATCHTM [46] programs, which utilize a large library of position weighted matrices for TFBSs, and the putative binding site is located. Recently, many deep learning approaches have been developed to predict sequence specificity with more accuracy [47,48]. Figure 4 depicts one of these approaches named DeepSNR that uses convolution-de-convolution network and a deep learning algorithm to predict the TFBS.

The concept of implementing AI into drug discovery has been around for decades, however, an article in the *New York Times* in recent years made it of public interest wherein a deep learning network won a quantitative structure activity relationship (QSAR) machine-learning challenge in drug discovery hosted by Merck [49]. Thus far, various models have been developed to precisely predict new molecular entities as plausible therapeutic scaffolds [50,51,52]. In a recent study, Sanchez-Lengling et al. [53] successfully predicted drug-like molecules which coincided with FDA-approved drugs using their developed framework based on objective-reinforced generative adversarial networks (ORGAN). Olivercona et al. [54] developed a sequence-based generative model for molecular de novo design and have illustrated its application in predicting molecules with specified desirable properties. Another strategy employed by this group includes various types of autoencoder models that rendered new structures that were predicted to be active against dopamine type receptor 2 [55,56,57,58]. Even pharmaceutical giants such as Bayer Healthcare and Roche have attributed their recent success in developing optimized pharmacophores to computer-assisted drug design technologies [59,60]. A thorough review on this topic could be found elsewhere [61,62]. So far AI has been successfully implemented only to retrospectively generate de novo structures using already existing data set that encompasses known bioactive compounds and biological targets. However, in a recent study Merk et al. [63] have demonstrated the applicability of AI in delivering novel synthetically feasible bioactive compounds for the first time. In this prospective study, they have aptly modified their model to precisely recognize retinoid X and proliferator-activated receptor agonists and further synthesized and assayed the de novo generated compounds that altered receptor activity in cell-based assay. 

**Figure 5:**
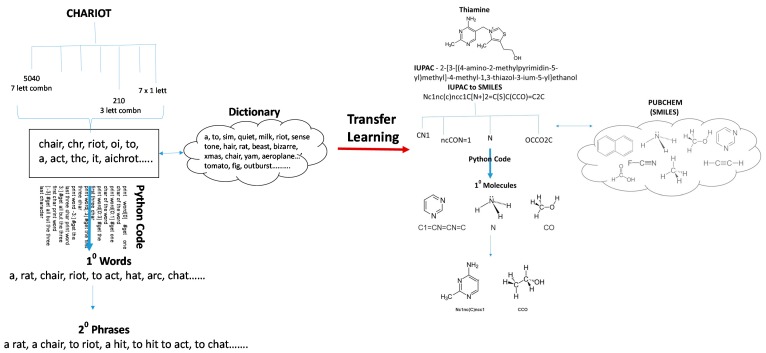
Transfer learning in medicinal chemistry. In this hypothetical example, an algorithm used to construct words from the English language character string CHARIOT, and subsequently phrases using those words, has been repurposed to compose small molecules from thiamine and then build larger molecules from those smaller molecules. These molecules could represent novel drug-like candidates or even metabolites of thiamine.

Transfer learning is a machine learning method where a model developed for a task is reused as the starting point for a model on a second task [64]. Algorithms used in speech recognition and language processing can be transferred to medicinal chemistry. In the hypothetical example shown in Figure 5, a Python-coded pattern matching algorithm has been used to first extract words from “CHARIOT”. While some or all the letters in CHARIOT can be reorganized a myriad number of ways, the pattern matching algorithm references a dictionary and only character combinations that make known words are retained Subsequently, one can employ a sentence-generating algorithm to build phrases and even sentences from these words. Such an algorithm can be transferred to an exercise in medicinal chemistry. In this example, the International Union of Pure and Applied Chemistry (IUPAC) name for thiamine is first converted to a SMILES nomenclature. The simplified molecular-input line-entry system (SMILES) is a specification in form of a line notation for describing the structure of chemical species using short ASCII strings. SMILES strings can be imported by most molecule editors for conversion back into two-dimensional drawings or three-dimensional models of the molecules. While a myriad number of smaller SMILES strings, connoting molecules and impossible molecules, a Python-coded pattern-matching algorithm (run against a known database of chemicals such as PUBCHEM) is used to retain known smaller molecules and then build larger molecules based on existing rules in organic chemistry. While the machine/computer generated 2° generation molecules could represent drug-like candidates, in this example, the 1° and 2° generation small molecules could just as easily represent Phase 1 and Phase 2 metabolites, respectively, of thiamine.

Another method to facilitate the synthetic process of novel drug candidates involves a continuous loop of self-learning in which, initially, it takes advantage of an existing data set of known bioactive compounds and is expected to produce therapeutically relevant structures [65]. When it comes to certain sequential machine learning tasks, such as speech recognition, recurrent neural networks (RNNs) are reaching levels of predictive accuracy, time and time again, that no other algorithm can match. Most artificial neural networks, such as feedforward neural networks, have no memory of the input they received just one moment ago. Recurrent networks, on the other hand, do remember what they have just encountered, and at a remarkably sophisticated level especially RNNs incorporating long short-term memory (LSTM) [66]. Medicinal chemists are exploring to what extent an RNN with LSTM cells can figure out sensible chemical rules and generate synthetically feasible molecules after being trained on existing compounds encoded as SMILES. The networks can to a high extent generate novel, but chemically sensible molecules. The properties of the molecules are tuned by training on two different datasets consisting of fragment-like molecules and drug-like molecules. The produced molecules and the training databases have very similar distributions of molecular weight, calculated logP, number of hydrogen bond acceptors and donors, number of rotatable bonds and topological polar surface area when compared to their respective training sets. The compounds are for the most cases synthetically feasible as assessed with the synthetic accessibility or SA score and Wiley ChemPlanner. 

Akin to many scientific discoveries, AI in drug discovery has harbored healthy skepticism among the medicinal chemists. It is yet to be tested how AI can tackle the challenge of imbibing natural products to build synthetically feasible de novo designs as effective pharmacophores [67,68]. Also, the ventured chemical space has largely been small molecules; however, the advent of bio-conjugate therapies such as immunotherapy, RNAi therapeutics etc., where chemistry converges with biology is yet to be explored. In fact, converging the large pool of datasets starting from hypothesis to pharmacovigilance that encompasses multiple variables has never been easy. Most importantly, a single AI-projected drug candidate is yet to make it as approved therapeutics. It might still be a prudent idea to cross validate the AI projected de novo designs by experienced medicinal chemists prior to taking the onus of drug discovery. However, current advancement in the field holds the promise to resolve the issues in coming future and possibly make an everlasting impact in the realm of drug discovery.

## 5. Conclusions

The field of directed drug design and molecular therapy is set to grow exponentially, aided greatly by inroads made in omics analysis, precision medicine, big data capture and analysis, deep learning and AI. In fact, the marriage of AI-aided drug discovery and synthesis and precision medicine-aided drug application and patient management will revolutionize the field of medicine. It is not difficult to imagine that one day each patient will benefit from the use of smart designer drugs tailored to that individual. Hydrogel-based multi-drug depots could be implanted in patients with multiple comorbidities. Such a depot would only contain drugs tailored to the omics signature in that patient. The depot would deploy a particular drug only on demand—say a nephroprotective whose release is linked to an excursion in the patient’s serum creatinine (SCr). Furthermore, just as many of today’s smart phones having an inbuilt audio amplification mechanism in the setting of excess ambient noise or just as many cars’ headlights coming on and off as the vehicle enters and exits an underpass, respectively, the nephroprtective drug’s clearance could be tuned to a decrease in SCr by linking SCr with drug levels and the appropriate cytochrome P450. Without a doubt, we have just started scratching the surface of the AI and precision medicine revolutions in drug discovery, molecular medicine and patient management.
